# Oophoropexy for ovarian torsion: a new easier technique

**DOI:** 10.1186/s10397-017-1001-9

**Published:** 2017-05-09

**Authors:** Tamer A. Hosny

**Affiliations:** Department of Obstetrics and Gynecology, Alexandria University Hospital, 16A Mohamed Said Pasha street, San Stefano, Alexandria, 21411 Egypt

**Keywords:** Ovarian torsion, Oophoropexy, Laparoscopy, Ovarian hyperstimulation syndrome, Trocar site closure needle

## Abstract

**Background:**

Oophoropexy for ovarian torsion is easy to be done by many tools either suturing to the lateral pelvic wall, plication of the ovarian ligament or even fixation to the back of the uterus, but it is little bit difficult to do it for pregnant women with less manipulation.

**Objective:**

We propose that using trocar site closure needle can be easier and faster technique to do this. To assess the feasibility of using the trocar site closure needle to do oophoropexy in ovarian torsion and its possible applicability.

**Patients:**

Seven patients presented with ovarian torsion; four of them were pregnant at 7, 15, 19 and 20 weeks of gestation, two patients with ovarian hyperstimulation in IVF cycles and one adolescent patient with hemorrhagic cyst. They were diagnosed by clinical presentation and ultrasound with Doppler analysis, and confirmed by laparoscopy where they underwent detorsion and fixation of the ovary using the trocar site closure needle.

**Results:**

Follow up of all the cases after one week showed improvement of the symptoms and normal Doppler flow of the target ovary then after three weeks by ultrasonography which revealed normal Doppler flow in the previously torsioned ovary. Two pregnant women underwent cesarean delivery where the operated ovary was observed during the delivery and was normal in shape and freely mobile with no adhesions.

**Conclusion:**

We propose that this technique is easier, faster and more comfortable especially in ovarian torsion in pregnant women and torsion in hyperstimulated ovaries.

**Electronic supplementary material:**

The online version of this article (doi:10.1186/s10397-017-1001-9) contains supplementary material, which is available to authorized users.

## Background

Ovarian torsion occurs when the ovary rotates around the infundibulopelvic ligament and the ovarian ligament interfering with its blood supply, which may be partial or complete. It is one of the most common gynecologic emergencies in all age groups [1]. The primary risk factor for the ovarian torsion is the presence of a mass which may be either a physiologic cyst or a neoplasm [2–4].

The frequent presenting symptoms are acute onset of pelvic pain, nausea, vomiting, fever, and adnexal mass with or without abnormal genital tract bleeding [4, 5]. A high index of suspicion is required to make the diagnosis especially if there is a history of ovulation induction for treatment of infertility [6] or during pregnancy. Pelvic ultrasound is still the first-line image study for diagnosing a patient with suspected ovarian torsion. The sonographic findings that are associated with ovarian torsion are described in many studies [7, 8]. Diminished or absent ovarian vessel flow on two-dimensional, color, and three-dimensional Doppler ultrasound has been proposed as a test for ovarian torsion [9–12]. Direct visualization of the rotated ovary remains the confirmatory way to diagnose the torsion, and the laparoscopic approach is typically used also to evaluate the ovarian viability [13].

Ovarian conservation is the preferred approach for premenopausal women, and most ovaries should be considered potentially viable unless there is a high degree of certainty that the ovary is not viable due to the presence of necrotic tissue. The conservative management consists of detorsion of the ovary followed by cystectomy if a mass is present. As ovarian torsion may recur after detorsion [14, 15], unilateral or bilateral oophoropexy following detorsion may be performed to prevent recurrence [16].

## Patients and methods

**Fig. 1:**
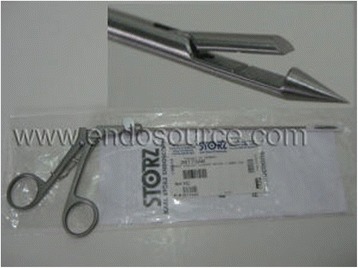
Closure site trocar needle



Seven patients presented with unilateral ovarian torsion to the emergency room in Alexandria University Hospital between November 2014 and May 2015; four of them were pregnant at 7, 15, 19, and 20 weeks of gestation; the torsioned ovaries were hyperstimulated in those pregnant women at 7, 15, and 20 weeks of gestation while there was an ovarian cyst in the pregnant woman at 19 weeks of gestation. Two patients had ovarian hyperstimulation in IVF cycles and one adolescent patient had a hemorrhagic cyst. They were diagnosed by clinical presentation and ultrasound with Doppler analysis and confirmed by conventional laparoscopy (Additional file 1: Video 1), where they underwent detorsion and fixation of the ovary using the trocar site closure needle (Fig. 1) at the same setting.

**Additional file 1: Video 1.**. (MP4 27.6 mb)


## Technique

This idea is mainly to present an easy technique for emergency procedure.

- ➢ Laparoscopic entry after pneumoperitoneum insufflation via Veress needle at the umbilicus or Palmer point for the pregnant women. The camera was placed in a 10-mm trocar at the umbilicus or in a 5-mm trocar at the Palmer point for the pregnant women at 19 and 20 weeks of gestation.
- ➢ Using two ancillary trocars, detorsion was performed followed by ovarian bivalving or cystectomy in cases of ovarian cysts
- ➢ Fixation of the ovary by transfixing the trocar site closure needle with absorbable vicryl 2-0 suture through the ovary then picking the suture from another transfixing point through the ovary then tying the suture out around the sheath (Additional file 2: Video 2).

**Fig. 2:**
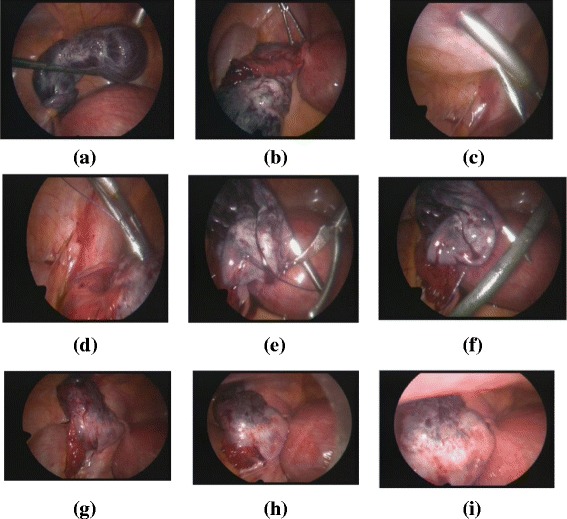
**a** Twisted right ovary. **b** Detorsion. **c** Site of entrance for trocar site needle. **d** Entry through the ovary. **e** Holding threads after transfixing the ovary. **f** Second entry. **g** Fixing the ovary to the abdominal wall. **h** During deflation. **i** After complete deflation

**Additional file 2: Video 2.**. (MP4 32.6 mb)
- ➢ The technique is illustrated in Fig. 2.

## Results

**Fig. 3:**
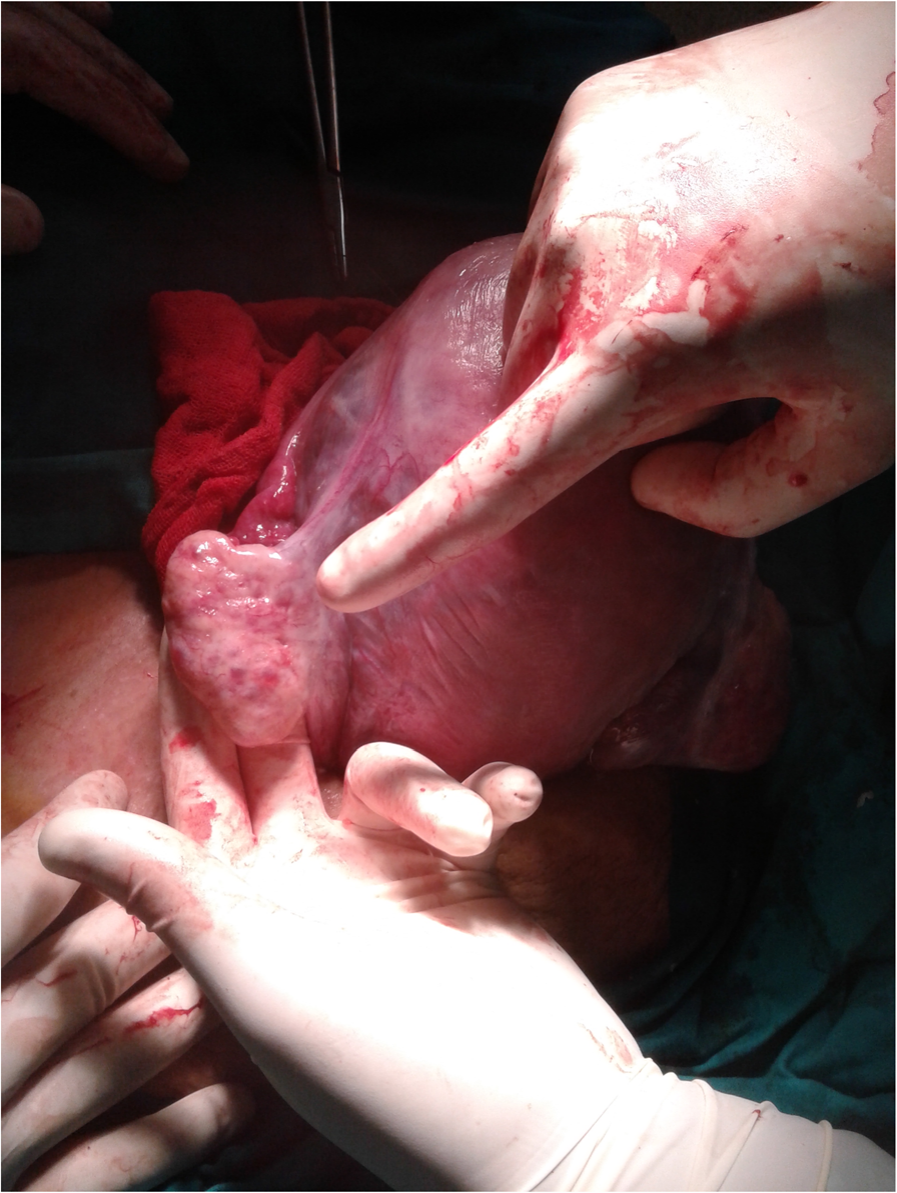
Picture of ovary during cesarean section

Follow-up of all the cases after 1 week showed improvement of the symptoms and then normal Doppler flow of the target ovary after 3 weeks by ultrasonography which revealed normal Doppler flow in the previously torsioned ovary. Two pregnant women underwent cesarean delivery where the operated ovary was observed during the delivery and was normal in shape and freely mobile with no adhesions (Fig. 3).

## Discussion

Although oophoropexy for ovarian torsion is debatable situation, retorsion may occur [14, 15]. Oophoropexy for ovarian torsion is emergency procedure, if we compare the most accepted way of oophoropexy by ovarian ligament placation I think it needs more training for suturing by laparscopy and it will be very difficult in cases of pregnant uterus, so we propose that this technique may be helpful although comparative study must be done between ovarian ligament placation and this technique illustrated, but limited number of cases of ovarian torsion, as it is one of the rare emergency situation.

## Conclusions

We propose that this technique is easier, faster, and more comfortable especially in ovarian torsion in pregnant women and torsion in hyperstimulated ovaries.
